# Single-case experimental designs for bumetanide across neurodevelopmental disorders: BUDDI protocol

**DOI:** 10.1186/s12888-022-04033-8

**Published:** 2022-07-07

**Authors:** Lisa Geertjens, Gianina Cristian, Eva Haspels, Jennifer Ramautar, Gert Jan van der Wilt, Matthijs Verhage, Hilgo Bruining

**Affiliations:** 1 N=You Neurodevelopmental Precision Center, Amsterdam Neuroscience, Amsterdam Reproduction and Development, Amsterdam UMC, Meibergdreef 5, 1105 AZ Amsterdam, The Netherlands; 2 Child and Adolescent Psychiatry and Psychosocial Care, Emma Children’s Hospital, Amsterdam UMC, Vrije Universiteit Amsterdam, De Boelelaan 1105, 1081 HV Amsterdam, the Netherlands; 3 Department of Health Evidence, Radboud University Medical Center, 6500 HB Nijmegen, The Netherlands; 4 Levvel, Center for Child and Adolescent Psychiatry, Meibergdreef 5, 1105 AZ Amsterdam, The Netherlands; 5 Department of Human Genetics, Center for Neurogenomics and Cognitive Research (CNCR), Amsterdam UMC-location VUmc, De Boelelaan 1105, 1081 HV Amsterdam, The Netherlands; 6 Department of Functional Genomics, Center for Neurogenomics and Cognitive Research (CNCR), VU University Amsterdam and Amsterdam UMC-location VUmc, de Boelelaan 1085, 1081 HV Amsterdam, The Netherlands

**Keywords:** Bumetanide, Autism spectrum disorder, Neurodevelopmental disorders, Children

## Abstract

**Background:**

Bumetanide is a selective NKCC1 chloride importer antagonist which is being repurposed as a mechanism-based treatment for neurodevelopmental disorders (NDDs). Due to their specific actions, these kinds of interventions will only be effective in particular subsets of patients. To anticipate stratified application, we recently completed three bumetanide trials each focusing on different stratification strategies with the additional objective of deriving the most optimal endpoints. Here we publish the protocol of the post-trial access combined cohort study to confirm previous effects and stratification strategies in the trial cohorts and in new participants.

**Method/design:**

Participants of the three previous cohorts and a new cohort will be subjected to 6 months bumetanide treatment using multiple baseline Single Case Experimental Designs. The primary outcome is the change, relative to baseline, in a set of patient reported outcome measures focused on direct and indirect effects of sensory processing difficulties. Secondary outcome measures include the conventional questionnaires ‘social responsiveness scale’, ‘repetitive behavior scale’, ‘sensory profile’ and ‘aberrant behavior scale’. Resting-state EEG measurements will be performed at several time-points including at Tmax after the first administration. Assessment of cognitive endpoints will be conducted using the novel Emma Tool box, an in-house designed battery of computerized tests to measure neurocognitive functions in children.

**Discussion:**

This study aims to replicate previously shown effects of bumetanide in NDD subpopulations, validate a recently proposed treatment prediction effect methodology and refine endpoint measurements.

**Trial registration:**

EudraCT: 2020–002196-35, registered 16 November 2020, https://www.clinicaltrialsregister.eu/ctr-search/trial/2020-002196-35/NL

## Background

Neurodevelopmental disorders (NDDs) are heterogeneous conditions grouped by the DSM-5 as attention deficit hyperactivity disorder (ADHD), autism spectrum disorder (ASD), intellectual disability and learning disorders [1]. The more severe forms often require medicinal interventions but options are currently restricted to symptom suppressing medication. Whereas for ADHD multiple stimulant drugs are registered, for ASD no medication is registered to improve the core defining features but drugs are often prescribed to mitigate associated symptoms such as depression, hyperactivity and irritability.

The advent of genetic animal models of neurodevelopmental conditions has led to the identification of possible mechanism-based treatments, most notably for ASD. One of the most studied options is selective Na^+^-K^+^-2Cl^−^ (NKCC1) antagonist bumetanide. Bumetanide is a registered loop diuretic that has been used for almost 50 years in adults and children with a variety of nephrological and cardiac conditions. Bumetanide has a mild side effect profile with diuretic effects such as electrolyte imbalance and hypokalemia that can be safely monitored when kidney function is normal [2, 3]. Blocking NKCC1 chloride import in the brain can lower chloride concentrations and potentially reinstate GABAergic inhibition. In normal development a developmental sequence occurs around birth, which is characterized by dramatic decrease in chloride concentration in neuronal cells. This maturational downregulation of chloride levels causes a shift in the so-called polarity of GABAergic transmission from excitatory (depolarizing) to inhibitory (hyperpolarizing): as referred to as the GABA-shift. The GABA shift is mediated predominantly by a change in the expression of two chloride co-transporters: the Na-K-2Cl cotransporter isoform 1 (NKCC1) importer and K-Cl cotransporter isoform 2 (KCC2) exporter [4, 5], hence the potential for bumetanide to restore inhibitory GABA signaling. Since GABAergic inhibition has an important role in maintaining E/I balance for proper neuronal growth, and synapse and circuit development, alterations in polarity may have wide-ranging consequences. Indeed, in model studies for ASD [6–8], epilepsy [9], Rett syndrome [10] and Down syndrome [11], the GABA shift was found to be abolished and excitatory effects of GABAergic signaling were established.

These findings lead to the initiation of bumetanide trials in ASD and several genetic disorders, with varying results [6–8]. This is in our opinion, in part the result of ignoring etiological heterogeneity of NDDs, which is likely to result in mechanism-based options not fulfilling a one-size-fits application (as opposed to symptom suppressing treatments). As such, we argue that these treatments will only be effective in a subset of patients with NDDs [2, 12]. Accordingly, we developed a set of trials testing different behavioral neurophysiological and genetic stratifications to evaluate efficacy across different diagnostic classes and to develop strategies for more successful application:

‘Bumetanide for autism medication and biomarker study’ (BAMBI), to replicate effectiveness in ASD on core symptomology and to develop electro-encephalogram (EEG) and cognitive stratification and prediction markers.

‘Bumetanide for the autism spectrum clinical effectiveness trial’ (BASCET), to test effectiveness in a cohort stratified by the presence of sensory reactivity problems across NDDs (ASD, ADHD, epilepsy).

‘Bumetanide for ameliorate tuberous sclerosis complex (TSC) hyper-excitable behaviors’ (BATSCH)’, an open label trial in children stratified by a genetic disorder with previously suggested efficacy of bumetanide.

In these trials, we evaluated multiple outcome levels including: behavior, cognition and neurophysiological changes using questionnaires, neurocognitive testing and resting-state EEG and event-related (ERP) markers, see Tables 1 and 2. The resting-state EEG markers focused on measuring effects on excitation-inhibition (E/I) ratios in line with the putative effect of bumetanide on GABAergic transmission [13]. The main findings included: 1) A superior effect of bumetanide on repetitive behavior in ASD (BAMBI) and TSC (BATSCH) [14, 15], 2) A significant effect of bumetanide on aberrant behaviors across disorders (BASCET and BATSCH), and 3) Enhanced power and excitation-inhibition ratios [13] only in the bumetanide treated group in the BAMBI trial. From the EEG effects, we developed an initial prediction algorithm by incorporating EEG biomarkers and clinical severity scores (RBS-r) [16].

**Table 1:** Details of BAMBI, BASCET and BATSCH cohorts

	BAMBI	BASCET	BATSCH
**Trial design**	RCT	RCT	Open label
**Cohort**	Unmedicated ASD	Children with sensory processing difficulties and ADHD/ASD or epilepsy	Children with TSC and behavioral problems
**Age (years)**	7–15	7–15	5–18
**n**	92	50	15
**IQ (mean, (SD))**	101,0 (20,4)	99,2 (24,2)	66,7 (23,9)

*ADHD* attention deficit hyperactivity disorder, *ASD* autism spectrum disorder, *RCT* randomized controlled trial, *TSC* tuberous sclerosis complex

**Table 2:** Outcomes of BAMBI, BASCET and BATSCH trials

Domain	Outcome measure	BAMBI	BASCET	BATSCH
**Behavioral outcomes**				
**Social communication and interaction**	SRS-2 (subscales social awareness, social cognition, social communication, social motivation)	X^a^	X	X
**Restricted, repetitive patterns of behavior**	SRS-2 (subscale mannerisms)	X^a^	X	X
	RBS-r	X	X	X
**Aberrant behavior**	ABC	X	X^a^	X^a^
**Sensitivity to sensory stimuli and Sensory stimulation tolerance**	SP-NL	X	X	X
	SP-SC		X	X
	HSP	X	X	
**Quality of life and social improvement**				
**Quality of life**	QoL	X	X	X
	EQ-5D-5L	X	X	X
	EQ-5D-Y	X	X	X
	pedsQL		X	X
**Executive functions**	TRF	X		X
	BRIEF	X		X
**Economic evaluation**	iPCQ	X		X
	TIC-P	X		X
**Disease specific outcomes**				
**TSC**	TAND checklist			X
**Epilepsy**	Epilepsy variables		X	
**Cognitive measurements**				
**Working memory**	Digit span of the WISC-III, spatial span of the Wechsler Nonverbal scale of ability	X		X
**Memory**	Rey auditory verbal learning test, rey visual design learning test	X		X
**Semantic memory**	Recalling sentences subtest of the clinical evaluation of language fundamentals	X		X
**Baseline response speed**	ANT	X		X
**Prepotent response inhibition**	Go-nogo task and condition two of the auditory and visual shifting set tasks of the ANT	X		X
**Attentional flexibility**	Condition three of the auditory and visual shifting set tasks of the ANT	X		X
**Neurophysiological outcomes**				
**rsEEG**	Alpha relative and absolute power, central frequency, detrended fluctuation analysis and excitation/inhibition ratio	X		X
**ERP**	PPI paradigm, P50 paradigm, mismatch negativity paradigm, selective attention paradigm	X		X

Primary outcome measures are marked with^a^ *ABC* Aberrant behavior scale, *ANT* Amsterdam neuropsychological task battery, *BRIEF*: behavior rating inventory of executive function, *EQ-5D-5L* 5-level EuroQoL 5-dimensional questionnaire, *EQ-5D-Y* 5-level EuroQoL 5-dimensional questionnaire, youth version, ERP: event-related potential, *HSP* highly sensitive child or parent scale, *iPCQ* productivity cost questionnaire, *pedsQL* pediatric quality of life inventory, *QoL* quality of life, *RBS-r* repetitive behavior scale revised, *rsEEG* resting state electro-encephalogram, *SP-NL* sensory profile, Dutch version, *SP-SC* sensory profile, school companion, *SRS-2* social responsiveness scale, second edition, *TAND* tuberous sclerosis associated neuropsychiatric disorders, *TiC-P* Trimbos/iMTA questionnaire for Costs associated with Psychiatric Illness, *TRF* teacher report form, *TSC* tuberous sclerosis complex

Another important observation was the consistent improvement of several symptoms not captured by the conventional outcome scales. For instance, symptoms such as fatigue, irritability, energy and sleeping problems seemed responsive to bumetanide. To evaluate these symptoms, we have recently developed a ‘patient reported outcome’ (PRO) set that we chose as the primary endpoint in this post cohort study to serve as a potential more personalized method of outcome measuring (van Andel under review).

Here we present the follow-up trial protocol developed to replicate previous bumetanide effects, improve clinical endpoint selection and to validate the treatment prediction algorithm.

## Methods/design

The Bumetanide for developmental disorders (BUDDI) study is a post-trial access cohort using Single-Case Experimental Designs (SCEDs) testing bumetanide treatment during 6 months. This type of N-of-1 design is most appropriate for our post-trial access cohort as 1) N-of-1 designs involving placebo treatment periods may not be tolerated, as many participants have already participated in placebo-controlled experiments (i.e. BAMBI, BASCET) and 2) the washout data of BAMBI and BASCET trials suggested prolonged effects of bumetanide treatments, which cause difficulty in placebo versus treatment cross-over designs due to carry over effects. The study will be performed at the N=You neurodevelopmental Precision Center at the Emma Children’s Hospital in the Amsterdam University Medical Center (AUMC), the Netherlands. SCEDs are preferred over the conventional post-trial access design (open label), because of the goals of more individualized effect measurements and improvement of clinical end point selection.

### Design

We will use multiple baseline SCEDs (MBD) in which the intervention (bumetanide) is introduced sequentially to different patients with a baseline period ranging from 2 to 12 weeks. The rationale for this multiple baseline is that apart from clinical and response heterogeneity across individuals also symptoms per patient vary over time. In an MBD the variation in the baseline period (A phase) is compared to the variation during the intervention (B phase) on an individual level (i.e. the participant serves as his/her own control). Evidence of such an AB designs is based on demonstrating that the change in behavior only occurs during intervention.

### Study population

All participants that participated in the previous studies, as well as a new cohort with matching inclusion and exclusion criteria are eligible for this post-trial access study. See Table 3.

**Table 3:** In- and exclusion criteria BUDDI trial

Inclusion criteria	Exclusion criteria
1. Inclusion in BAMBI, BASCET or BATSCH trial; 2. Written informed consent Or 1. Males or females aged ≥7 years to ≤17 years; 2. One of the following: 3. Above clinical cut-off scores of altered sensory reactivity on the Sensory Profile and either a clinical ASD or ADHD diagnosis based on DSM-5 (or DSM-IV) or an epilepsy diagnosis, 4. Criteria met for autism on DSM-IV or V and Social Responsiveness Scale (SRS-2) 5. A history of behavioral problems combined with a definite diagnosis of TSC: either meeting criteria for clinical definite TSC, or a mutation identified in the TSC1 or TSC2 gene; 6. Written informed consent	1. Inability to comply with the protocol-specified procedures for the duration of the study, including treatment, blood sampling to control diuretic effects; a. This does not include inability to perform neurocognitive testing due to intellectual disability, as there is no minim IQ needed. 2. Presence of a severe medical or genetic disorder other than related to ASD, TSC or epilepsy; 3. Serious, unstable illnesses including, gastroenterological, respiratory, cardiovascular (arrhythmias, QT interval lengthening), endocrinologic, immunologic, hematologic disease, dehydration or hypotension, electrolyte disturbances (Na < 133 mmol/L, K < 3.5 mmol/L or Ca < 2.17 mmol/L (<13y) or < 2.2 mmol/L (>13y); 4. Renal insufficiency (CKD st2–5; estimated glomerular filtration rate < 90 ml/min/1.73 m2), congenital or acquired renal disease with decreased concentration capacity (tubulopathy, diabetes insipidus) and liver insufficiency interfering with excretion or metabolism of bumetanide; 5. Start of behavioral treatment during study; 6. Treatment with psychoactive medications, including antipsychotics and AEDs, **except** methylphenidate, is allowed if on a stable regime in terms of types and dosage from 2 months prior to the study to the end of the study; 7. Treatment with NSAIDS, aminoglycosides, digitalis, antihypertensive agents, indomethacin, probenecid, acetazolamide, Lithium, other diuretics (e.g., furosemide, hydrochlorothiazide), drugs known to have a nephrotoxic potential; 8. Documented history of hypersensitivity reaction to sulfonamide derivatives; 9. Body weight < 30 kg (for reason of dosing).

### Recruitment and screening

All previous participants of the BAMBI, BASCET and BATSCH trials will be contacted and informed about this post-trial access study. If interested and eligible, previous participants will receive verbal and written information about the study. We estimate that 50% of the patients that were enrolled in the three previous bumetanide trials will be eligible and motivated to be enrolled in the present study (i.e., 75 patients in total).

Participants of the new cohort will be recruited from the patient population referred to the N=You neurodevelopmental Precision Center at the Emma Children’s Hospital in the AUMC.

### Intervention and preparation of study drugs

The intervention constitutes of bumetanide tablets. Bumetanide will be provided at a starting dose of 0.5 mg twice daily and will be increased to the therapeutic dosage of 1.0 mg twice daily at day 7 if there are no signs of dehydration in all participants. In case of limited effects and side-effects and a weight > 45 kg dosage can be increased to 1.5 twice daily. Dose reductions to manage side effects will be allowed at any time. Tablets will be obtained via the research pharmacy of AUMC. Re-labeling will be prepared and applied according to local regulatory requirements (GMP annex 13 guidelines). Participants are instructed to return unused tablets to allow monitoring of drug adherence.

### Randomization

A randomization list of 115 baseline periods (2–12 weeks, with an equal distribution between the 11 intervals) is generated using the statistical program SPS. Upon signing informed consent, the participant receives the baseline period corresponding with the next open slot on the list.

### Outcomes and measurements

#### Primary outcome

The primary outcome is a set of patient reported outcome measures (PROMs) containing questions directly or indirectly related to sensory processing difficulties, which will be filled in by caretakers. We chose this outcome over more conventional outcome measures for three reasons. 1) it allows for more personalized method of outcome measuring, 2) the PROMs are selected based on the symptoms not captured by conventional outcome scales (see background), 3) A prerequisite for the primary outcome of a SCED is the frequent and repeated measurement of the target behavior in every phase to address the variability in that behavior during the baseline and the intervention phase. The selected PROM-set asks the respondent to reflect upon the last 7 days, whereas conventional questionnaires often ask for reflection upon a longer time scale, making them less suitable for a SCED design.

The effects on PROMs will be compared to main conventional endpoints used in the original trials (social responsiveness scale, second edition (SRS-2) [17], repetitive behavior scale revised (RBS-r) [18], aberrant behavior scale (ABC) [19] and sensory profile – Dutch version (SP-NL) [20]) as well as accompanying measurements of EEG and neurocognition to further establish effects on brain activity and functioning and to validate predictive markers of treatment response. Individual results will be aggregated to evaluate bumetanide efficacy on a group level.

#### Secondary outcomes

The secondary outcome measures are divided over three domains: the behavioral, the functional and the translational domain (see Table 4).

**Table 4:** Outcome measures

Domain	Outcome measure
**Primary outcome measures**	
**Behavioral domain**	
**Anxiety**	PROMIS Anxiety (v2.0) PROMIS Psychological stress experiences (v1.0)
**Mood problems**	PROMIS Depressive symptoms (v2.0) PROMIS Life satisfaction (v1.0)
**Sleep problems**	PROMIS Sleep-related impairment (v1.0) PROMIS Sleep disturbance (v1.0)
**Fatigue**	PROMIS Fatigue (v2.0)
**Physical complaints**	PROMIS Physical stress experiences (v1.0)
**Daily functioning and participation**	PROMIS Cognitive function (v.1.1), VABS
**Problems in social interaction and communication**	PROMIS Peer relationships (v2.0)
**Secondary outcome measures**	
**Behavioral domain**	
**Social communication and interaction**	SRS-2 (subscales social awareness, social cognition, social communication, social motivation)
**Restricted, repetitive patterns of behavior**	SRS-2 (subscale mannerisms) RBS-r
**Sensory stimulation tolerance**	SP-NL
**Sensitivity to sensory stimuli**	SP-NL
**Aberrant behavior**	ABC
**Functional domain**	
**Cognitive**	Emma-toolbox
**Neurophysiological**	rsEEG
**Translational domain**	
**Genetic**	Whole exome sequencing
**Cellular**	Assays on iPSC-derived neuronal models

*ABC* Aberrant behavior scale, *PROMIS* patient reported outcome measurement information system, *RBS-r* repetitive behavior scale revised, *rsEEG* resting state electro-encephalogram, *SP-NL* sensory profile, Dutch version, *SRS-2* social responsiveness scale, second edition, *VABS* Vineland scale of adaptive behavior

##### Behavioral domain

The behavioral domain focuses on clinical outcomes and constitutes of four questionnaires to evaluate core symptomatology. The scales used are consistent with those used in the previous trials: The SRS-2, RBS-r, ABC and SP-NL.

In addition, these questionnaires will be used to validate how well the PROM set captures classically defined core symptomatology.

##### Functional domain

The functional domain contains neurocognitive and neurophysiological measures. We will use the Emma Tool box, an in-house designed battery of computerized tests, to measure neurocognitive functions in children. Measures will be obtained at 3 time points (baseline and after three and 6 months of treatment). Individual change in domain scores will be analyzed.

We will perform resting state electroencephalography (EEG) to assess neurophysiological functioning. EEG will be recorded by using the 128 channels Magstim/EGI system. EEG data will be processed offline using the Neurophysiological biomarker toolbox (http://www.nbtwiki.net/). Similar to our previous trials we selected five biomarker algorithms that have proven sensitive to the ratio of excitation and inhibition in computational models of neuronal networks generating alpha-band oscillations to quantify EEG. These biomarkers include: Relative and absolute power, central frequency, detrended fluctuation analysis and excitation/inhibition ratio. EEGs will be obtained at baseline, Tmax (1,5 h after first dose) and shall be repeated on a monthly basis.

##### Translational domain

The translational domain focusses on methods for future translation of (emerging) disease mechanisms and the development of more personalized therapies.

One entry point for personalized therapies are studies in animal models with causal genetic variants for NDDs. Hence, we will perform genetic testing via whole exome sequencing.

In addition, induced pluripotent stem-cell (iPSC) based model systems provide the opportunity to examine disease mechanisms in patient-own neurons and provide the opportunity to test personalized treatment options. Accordingly, we will perform assays with iPSC derived neuronal models.

#### Safety procedures

Safety will be assessed by the research team under supervision of a child psychiatrist and if necessary, a pediatric nephrologist. The assessment includes checks for the use of other medications, side effects and adverse events. In addition, physical examinations and blood and urine laboratory tests will be performed. See appendix 1 for a schematic overview of the examinations.

Oral potassium supplementation at a dose of 0.25 mmol/kg twice daily will be prescribed via custom pharmacy to all participants in order to avoid hypokalemia. Additionally, adjustments in the dosage of bumetanide are allowed to manage hypokalemia and/or side effects.

### Statistics

#### Power calculations

Without using any kind of data simulation, we estimate that 50% of the patients that were enrolled in the three previous bumetanide randomized controlled trials (RCTs) will be eligible and motivated to be enrolled in the present study (i.e. 75 patients). To estimate sample size requirements and type I and II error magnitudes of a statistical test in a SCED design, the outcome measure that is assessed on the most frequent basis should be used, in our case the PROMs. Since this measure has not been included in the previous RCTs conducted in our patient cohort, we cannot rely on effect size estimates for this particular outcome. As such, due to availability we based the power computations on outcome measures used in the BAMBI study. Specifically, we established the minimal sample size required to detect a significant effect on the secondary BAMBI outcome, improvement on repetitive behavior scale, on which previously, a significant effect was observed.

For this purpose we used an online tool suitable for multiple baseline single case designs [21]. The tool tests a statistically significant effect using a non-parametric, randomization test (see *Data Analysis*) and the following inputs: effect size to be estimated, number of permutations, number of patients, number of outcome measurements.

In a randomization test, an effect estimate is obtained by drawing a large number of samples from a “randomization distribution”. To overcome the computational difficulties arising from drawing all the possible samples, using Monte Carlo sampling we approximate as accurately as possible the result we would get when drawing all samples. To ensure a compromise between accuracy and computational feasibility, our power simulation uses repeated draws, namely 5 Monte Carlo chains, each drawing 100 samples.

We tested whether, given the current experimental setup, we are able to detect an effect comparable in size to the one obtained in the BAMBI study. For RBS-r, the outcome variable previously shown to be significantly impacted, the effect size of the treatment was estimated to be *d =* 0.373 (80% C.I. 0.225–0.529), using Cohen’s *d* for dependent samples. The simulations showed that an effect size *d =* 0.4 can be detected with a relatively high power (0.71 when *N =* 40 and 0.73 when *N =* 50), in the absence of autocorrelation. In practice, measurements which are close in time are related, thus outcome scores at one time point can predict scores at another time point (autocorrelation or serial dependence). With larger sample sizes (*N =* 60), the power to detect the same effect remains fairly high (0.69) even in the presence of low-medium (*r =* 0.3) autocorrelation.

Thus, *N =* 40 is an acceptable minimum sample size to be able to detect the BAMBI effect in a no-autocorrelation scenario and *N =* 60, a minimum sample size in the presence of low-to-medium autocorrelation. As we intend to enroll a minimum of 75 patients from previous cohorts and a minimum of 40 patients in the new cohort, we expect to have sufficient power to detect a comparable treatment effect.

#### Data analysis

For the data analysis visual inspection of the data and statistical inference using (interrupted) time series analysis and randomization tests will be performed.

##### Visual inspection using 2SD-band method

First, the PROMs will be plotted as its own time-series for visual inspection using the 2-SD band method, as described in Hoogeboom et al. [22]. The 2-SD band will be calculated from the baseline data and graphed from the baseline through the intervention and post-intervention phase. If two or more successive data points in the intervention or post-intervention phase fall outside the 2 SDs bandwidth, the result will be considered significant on an individual basis. As autocorrelation can bias the visual inspection, we will check our data in each phase for serial dependence using the lag-1 method. If data are found to be significantly correlated, we will transform the data using a moving-average transformation.

##### Parametric methods: (interrupted) time series

Single subject measurements are graphed over time in a series based on which we can predict measurements at future time points for that subject.

Due to repeated measurements, the series may exhibit patterns such as autocorrelation, seasonality and trends not explained by the intervention (non-stationarity). Failing to account for said patterns can lead to erroneous effect predictions. Based on visual inspection of the series and residuals, special regression models “*ARIMA”* (*AR* auto-regressive, *I* integrated, *MA* moving-average) can be tailored to account for said patterns and get more accurate predictions. To obtain group effects, the single-case effect predictions will be aggregated using meta-analysis.

Given our study design we are interested in comparing the (predicted) outcome evolution based on the baseline measurements, to the (observed) evolution based on post- intervention measurements. To that aim we use interrupted time series analysis (ITSA), an extension of classical time series analysis [23].

##### Non-parametric methods: randomization tests

If the SCED involves a small number of participants, parametric assumptions of data normality and homogeneity of variance might not be met, in which case, tests which do not make parametric assumptions might be more suitable, i.e. Koehler and Levin’s randomization test [24].

The null hypothesis of the randomization test is that, for all possible permutations, the mean difference between baseline and intervention is the same.

##### Selection bias

There is a likelihood of (patient-induced) selection bias, as it is more likely that previous participants from the responder groups will participate in this post trial access study. Accordingly, there will probably be a larger group of responders than non- responders.

We propose two methods to address this: controlling for covariates associated with selection (modelling-based) and inverse probability weighting. In the first approach, we will include a covariate describing previous responder status and check for significance. The second approach involves computing the probability of selection for each responder category and assigning a weight to each subgroup inverse to the selection probability.

#### Minimal clinically important difference (MCID)

Another aspect we wish to address is what constitutes a (non)responder. To determine the MCID for our target outcome scales, we intend to use comparisons of care-taker assessments in parent-to-parent conversations (anchor-based method).

#### Development of new predictors

The newly developed prediction algorithm based on EEG and clinical features will be evaluated and further developed [16]. Analyses will be performed using both regression analyses and supervised machine learning.

## Discussion

In conclusion, the BUDDI post trial access study will offer the opportunity to replicate individual and stratified group level effects of bumetanide, to improve clinical endpoint selection and to validate an EEG based treatment prediction algorithm. Forthcoming findings may enhance the applicability of bumetanide in heterogeneous NDD populations.

## Data Availability

Not applicable.

## Appendix 1

### Schematic overview of assessments

Below a schematic overview of assessments and safety procedures can be found.

Baseline parameters include: physical examination, extended blood analysis and urine analysis.

Blood analysis includes: sodium, potassium, chloride, uric acid, urea, creatinine, glucose, estimated glomerular filtration rate and total protein; The extended blood analysis includes alanine transaminase, asparate transaminase, gamma-glutamyltransferase, alkaline phosphatase and whole blood count.

Urine analysis includes: osmolarity, sodium, potassium, chloride, calcium, protein, creatinine, uric acid, microalbuminuria.

Physical examination includes: general appearance, weight, height, sitting and standing blood pressure, pulse rate and inspection of the skin, mouth and pharynx.

## Appendix 2

### Protocol revision chronology

Protocol version: 5.

Protocol amendment number: 2.

Issue date: 16-04-2021.

Author(s): H. Bruining, C. Van der Wit, B. Stunnenberg, M. Konings, A. Bouts, J Ramautar, G. Cristian, L. Geertjens.

Revision chronology:

**Table:**

Date	Protocol version
Protocol version 3, 20-08-2020	Original protocol
Protocol version 4, 29-01-2021	Version 4, Amendment 01 Primary reason for amendment: implementation of standard potassium supplementation
Protocol version 5, 16-04-2021	Version 5: Amendment 02 Primary reason for amendment: Inclusion of a new cohort
**only protocol versions approved by the medical ethical committee are included in the revision chronology*	

## Appendix 3

Table 5

**Table 5:** Trial registration data set

Data category	Information
Primary registry and trial identifying number	EudraCT database: 2020–002196-35
Date of registration in primary registry	16th November 2020
Secondary identifying numbers	NA
Source(s) of monetary or material support	Dutch National research agenda, the Netherlands
Primary sponsor	Amsterdam UMC, location VUmc
Secondary sponsor(s)	Na
Contact for public queries	*H. Bruining (MD, phd)* n.is.you@amsterdamumc.nl
Contact for scientific queries	*H. Bruining (MD, phd)* n.is.you@amsterdamumc.nl
Public title	Bumetanide for developmental disorders
Scientific title	Post trial access cohort bumetanide for developmental disorders
Countries of recruitment	Netherlands
Health condition(s) or problem(s) studied	Neurodevelopmental disorders, autism spectrum disorder, attention deficit hyperactivity disorder (ADHD), learning disorders
Intervention(s)	Bumetanide bidaily 0.5–1.5 mg
Key inclusion and exclusion criteria	Participation in previous bumetanide trials by H. Bruining (BAMBI, BASCET, BATSCH) *or* Ages eligible for study: 7–17 Sexes eligible for study: both Accepts healthy volunteers: no Inclusion criteria: One of the following: 1) Above clinical cut-off scores of altered sensory reactivity on the Sensory Profile and either a clinical ASD or ADHD diagnosis based on DSM-5 (or DSM-IV) or an epilepsy diagnosis, 2) Criteria met for autism on DSM-IV or V and Social Responsiveness Scale (SRS) 3) A history of behavioral problems combined with a definite diagnosis of TSC: either meeting criteria for clinical definite TSC, or a mutation identified in the TSC1 or TSC2 gene; Exclusion criteria: 1) inability to comply with study protocol, 2) presence of severe medical or genetic disorder other than related to ASD, TSC or epilepsy, 3) renal insufficiency, 4) start of behavioral treatment during study, 5) treatment with methylphenidate, NSAIDs, aminoglycoside, digitalis, antihypertensive agents, indomethacin, probenecid, acetazolamide, lithium or other diuretics, 6) history of hypersensitivity to sulfonamide derivatives 7) body weight < 30 kg
Study type	Intervention model: open label post-trial access
	Primary purpose: treatment
	Phase II
Date of first enrolment	December 2020
Target sample size	115
Recruitment status	Recruiting
Primary outcome(s)	Set of parent proxy PROMIS questionnaires: physical stress experience, psychological stress experience, sleep disturbances, sleep-related impairment, cognitive function, anxiety, fatigue, peer relationships, life satisfaction, depressive symptoms
Key secondary outcomes	Conventional questionnaires: SRS, RBS, ABC, SP-NL
	Resting-state EEG
	Neurocognitive testing (using in-house Emma Toolbox)

## Abbreviations

ABC: Aberrant behavior scale
ADHD: Attention deficit hyperactivity disorder
ASD: Autism spectrum disorder
BAMBI: Bumetanide for autism medication and biomarker study
BASCET: Bumetanide for the autism spectrum clinical effectiveness trial
BATSCH: Bumetanide for ameliorate tuberous sclerosis complex hyper-excitable behavior
BRIEF: Behavior rating inventory of executive function
BUDDI: Bumetanide for developmental disorders
EEG: Electro-enchephalogram
EQ-5D-5L: 5-level EuroQoL 5-dimensional questionnaire
EQ-5D-Y: 5-level EuroQoL 5-dimensional questionnaire, youth version
ERP: Event-related potential
HSP: Highly sensitive child or parent scale
iPCQ: Productivity cost questionnaire
iPSC: Induced pluripotent stem cell
KCC2: K-Cl cotransporter isoform 2
MBD: Multiple baseline SCEDs
MCID: Minimal clinically important difference
NDDs: Neurodevelopmental disorders
NKCC1: Na-K-2Cl cotransporter isoform 1
pedsQL: Pediatric quality of life inventory
PROM: Patient reported outcome measure
PROMIS: Patient reported outcome measurement information system
QoL: Quality of life
RBS-r: Repetitive behavior scale revised
RCT: Randomized controlled trial
rsEEG: Resting state electro-encephalogram
SCED: Single-case experimental design
SP-NL: Sensory profile. Dutch version
SP-SC: Sensory profile, school companion
SRS-2: Social responsiveness scale, second edition
TAND: Tuberous sclerosis associated neuropsychiatric disorders
TiC-P: Trimbos/iMTA questionnaire for Costs associated with Psychiatric Illness
TRF: Teacher report form
TSC: Tuberous sclerosis complex
